# Transfer of microorganisms to and from textiles in healthcare settings: a systematic review

**DOI:** 10.1017/ice.2025.10299

**Published:** 2025-12

**Authors:** Natalie Gassmann, Visar Vela, Walter Zingg, Aline Wolfensberger

**Affiliations:** 1 Department of Infectious Diseases and Hospital Epidemiology, University Hospital Zurich, Zurich, Switzerland; 2 Centre for Travel Medicine, WHO Collaborating Centre for Travellers’ Health, University of Zurich, Zurich, Switzerland; 3 Department of Pathology, University Hospital Basel, Basel, Switzerland; 4 Centre Suisse de Controle de Qualité (CSCQ), Geneva, Switzerland; 5 Institute for Implementation Science in Healthcare, University of Zurich, Zurich, Switzerland

## Abstract

**Objective:**

Microbial contamination of textiles in healthcare settings is common and hypothesized to contribute to pathogen transfer. This systematic literature review aims to summarize the current evidence on microorganism transfer to and from textiles in healthcare and on factors that influence transfer.

**Design:**

Systematic literature review.

**Methods:**

Cochrane, Medline/Ovid, EMBASE, and Web of Science were searched. Studies were included if the transfer experiment involved textiles as origin material or destination material, the transfer mechanism was described accurately, and transfer events were quantifiable. Results on transfer and factors associated with transfer were extracted.

**Results:**

We included 21 studies with 490 transfer experiments. Considerable heterogeneity in all relevant study variables resulted in a very broad range of reported transfer proportions, from less than 1% to up to 100%. Cotton was the most frequently studied textile (13 studies) while *Staphylococcus aureus* was the most frequent pathogen of interest (13 studies). Highest transfer proportions (85–100%) were reported in transfer experiments from solid surfaces to textiles by wiping. Very low transfer proportions (0.01–2.5%) were reported in transfer experiments from textiles to textiles by pressure. Moisture and friction were associated with higher transfer.

**Conclusions:**

This study highlights the wide range of microbial transfer quantity from and to textiles in healthcare, depending on transfer mechanism, moisture, and other factors. The findings can inform the design of infection prevention and control (IPC) practices in healthcare.

## Introduction

Transmission of pathogenic and multidrug-resistant microorganisms is relevant for patients because it can result in difficult-to-treat healthcare-associated infections (HAIs). The inanimate hospital environment is increasingly considered to contribute to in-hospital transmission.^1^ Textiles, including clothing, bedding, and curtains, are known to carry bacteria, viruses, and fungal organisms, and thus, can act as reservoirs and fomites.^2–5^ A recent report judged possible textile-associated outbreaks of microorganisms in healthcare settings to be relevant,^6^ but others considered the infection risk from textiles being low.^7^

The degree to which textiles act as fomites is still unclear. However, the potential role of textiles in healthcare-associated microbial transmission has sparked interest on fabrics with antimicrobial properties. Such textiles come with the promise of lowering the risk of healthcare-acquired infections by limiting textile-related transmission of microorganisms. Detailed knowledge on pathogen transfer by textiles can help infection prevention and control (IPC) to identify high-risk situations and to develop protocols for transfer mitigation, including the use of antimicrobial fabrics. The aim of this systematic review was to summarize the evidence on dimension and risk factors of pathogen transfer by textiles in healthcare.

## Methods

### Search strategy

We followed the guidelines of Preferred Reporting Items for Systematic Reviews and Meta-Analysis (PRISMA). The review was registered at the International Prospective Register of Systematic Reviews (PROSPERO) (No. CRD42021290377). Cochrane, Medline/Ovid, EMBASE, and Web of Science were searched for relevant papers (see **Appendix**: Search strategy). Studies meeting the inclusion criteria outlined below were analyzed, abstracted, and cross-referenced. Reference lists were screened for additional relevant studies. Studies in English, French, Italian, Spanish, and German, published before 24 August 2021 were included if an abstract in English was available.

### Selection criteria

The following criteria were applied for study inclusion:

Measurement of the transfer of microorganisms (bacteria, fungi, viruses, or parasites) from an origin material to a destination material, with either origin or destination material being a textile.

Textiles were made of fibers from either natural or synthetic sources and were produced by weaving, knitting, crocheting, knotting, tatting, felting, bonding, or braiding (including e.g. scrubs, isolation gowns, excluding e.g. toilet paper, plastic aprons).

The transfer mechanism was clearly described (including e.g. duration of contact, friction, pressure).

The microbial methods to contaminate, detect, and quantify microorganisms and to assess transmission probabilities were described in detail.

The transfer of microorganisms was quantified, allowing mathematical and statistical analysis.

The tested textiles can be used in healthcare.

We excluded studies investigating textiles treated with antimicrobial agents.

### Data extraction, data synthesis, and quality assessment

Two investigators (N.G. and A.W.) independently screened titles and abstracts, assessed full texts for eligibility, and extracted data. Discrepancies were resolved through discussion and joint review of the full text; studies were included if both investigators agreed that the inclusion criteria were met. Extracted data were compared to ensure consistency.

A total of 18 variables were extracted (see Appendix Table 1: Author; Year; Microorganism; Carrier Material of Microorganism; Origin Site; Inoculum at Origin Site; Destination Site; Environmental Conditions; Action Executed for Transfer; Number of Experiment Repetitions; Microbiological Sampling Method; Culturing Method; Controls to Assess Inoculum; Controls/Recovery Testing (efficiency of method in retrieving microorganisms from a surface); Transfer Proportion in %; Own Calculations to Assess % of Transfer; Results Extrapolated from Figure in %; and Significant Results Comparison).

Calculations (Equation 1) on transfer proportions were conducted if not reported by the study.
(1)

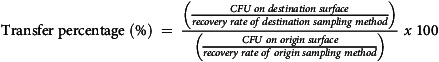




The level of analysis was the transfer experiment. In publications reporting different transfer mechanisms, data of all mechanisms were extracted. Last, statistically significant results of comparative tests assessing differences in transfer percentage between e.g. textile types, bacterial strains, transfer mechanism, or moisture were extracted.

Due to the heterogeneity of studies, with large variability of tested microorganisms, textiles, origin and destination materials, and sampling methods, we were not able to perform a meta-analysis. For descriptive analysis, studies were grouped based on shared origin and destination materials. Within each group, key findings were summarized, patterns identified, and discrepancies highlighted.

We applied a modified Downs and Black^8^ checklist for quality assessment (Appendix Table 2).

## Results

**Figure 1.**
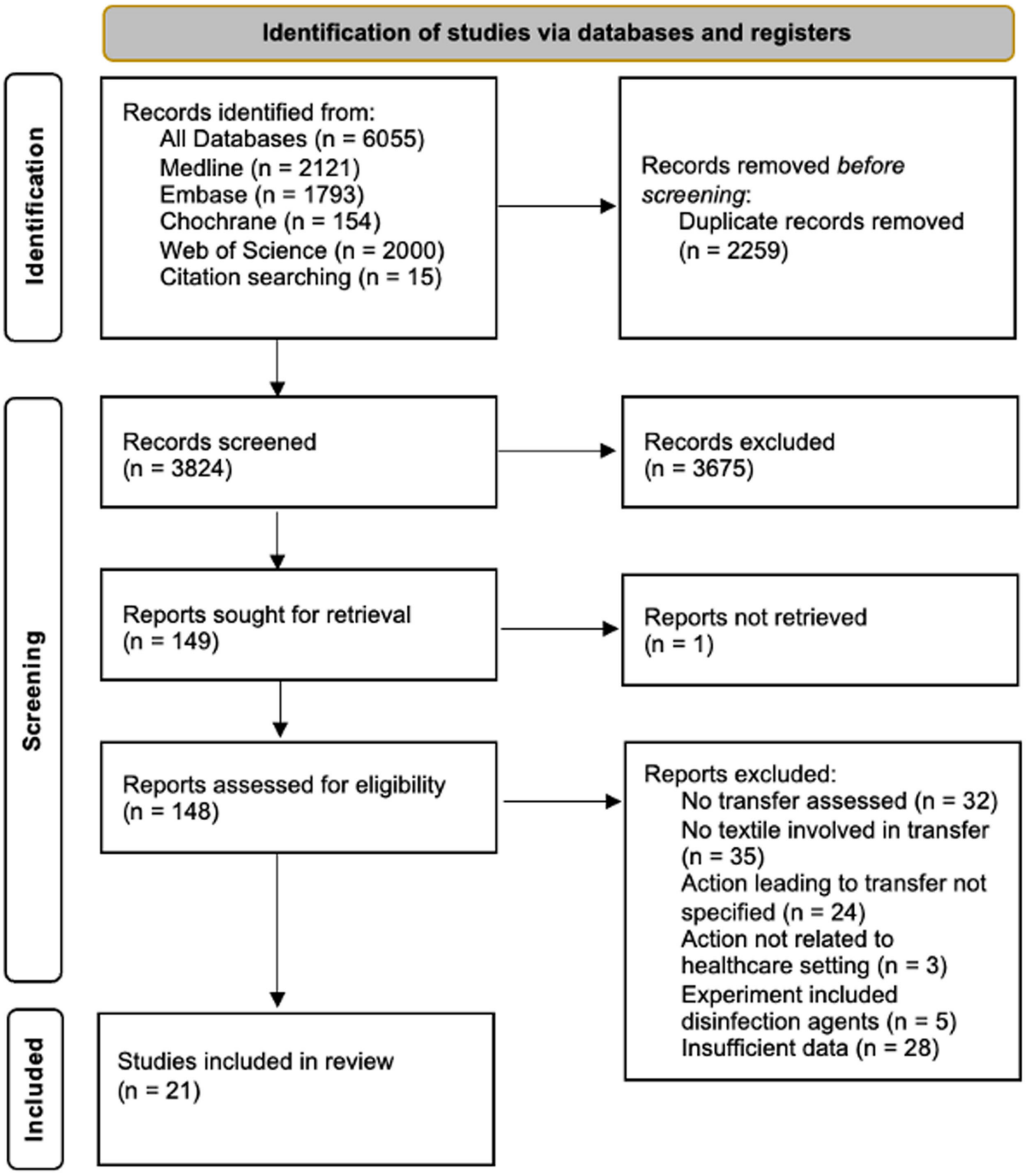
Study inclusion flow diagram. This diagram outlines the selection process of studies, from initial identification through final inclusion. It displays the number of records at each stage. Reasons for exclusions are noted for full-text screened studies. This diagram follows the standard PRISMA format.

After deduplication, 3824 titles and abstracts were screened. Of these, 148 studies were reviewed in full text (Figure 1). Finally, a total of 21 experimental studies met the eligibility criteria and were included for data analysis. They were published between 1970 and 2021 and reported 490 different transfer experiments. Table 1 summarizes the results of the 490 different experiments; Appendix Table 1 describes all variables and the results of each transfer experiment in detail.

**Table 1.** Summary overview of the included studies

Author (Year of publication) Quality Score	Microorganisms	Carrier material of pathogen	Origin material	Destination material	Transfer Mechanisms	Transfer Proportion	Recovery testing	Factors associated with transfer
Arinder et al. (2016)^21^ Score: 83%	*Staphylococcus aureus*	Difco™ D/E Neutralizing Broth	Vitro Skin	Cotton textile	Pressure of 2kPa for 10 seconds	6.7% +/− 16.5%	Not described	Humidity
Bartz et al. (2010)^22^ Score: 63%	*Escherichia coli*	Peptone water	Cotton textile	Stainless steel	Wiping (contact time or pressure not specified)	2.1% ^b^	Not described	–
Desai et al. (2011)^24^ Score: 92%	MRSA	Phosphate-buffered solution	Cotton textile	Pigskin	Adpression of pigskin with metal stamp for 3 seconds (pressure not specified)	1) <1–9%	Yes	–
Diab-Elschahawi et al. (2010)^28^ Score: 69%	*Staphylococcus aureus* *Escherichia coli*	Test soil (3% bovine serum albumin and 0.3% sheep erythrocytes)	Ceramic tiles	Microfiber textile (dry/moist, new/reprocessed) Cotton textile (dry/moist, new/reprocessed)	Wiping (contact time or pressure not specified)	96–99.9%^f^	Not described	Moisture New microfiber cloth (compared to reprocessed cloth) Reprocessed cotton cloth (compared to new cloth)
Edwards et al. (2017)^16^ Score: 73%	*Escherichia coli* *Staphylococcus aureus* *Enterococcus faecalis*	Phosphate-buffered solution with bovine serum albumin	Acrylic glass	Lyocell textile (untreated) Lyocell textile (C2F6 exposed^d^) Lyocell textile (O2 exposed) Polypropylene textile (untreated)	1) Pressure of 5 kPa for 10s 2) Wiping with 2kPa for 10s	1) 9–16%^a^ 2) 22–53%^a^	Not described	For *E. coli*: Polypropylene textile and C2F6-exposed lyocell textile (compared to untreated lyocell textile) E. coli (compared to other bacteria) Friction
Gerhardts et al. (2015)^20^ Score: 73%	*Staphylococcus aureus* *Streptococcus equi*	Artificial pus (solution with 1% macrophages containing 10 % fetal calf serum)	1) Artificial skin 2) Cotton textile 2) Polyester textile 2) Polyacrylic textile 2) Polyamide textile	1) Cotton textile 1) Polyester textile 1) Polyacrylic textile 1) Polyamide textile 2) Artificial skin	Pressure of 2.3 Pa for 2s a) with friction b) without friction	*S. aureus* 1a) 3.5–27.5%^b^ 1b) 1–3.5%^a^ *Streptococcus equi* 1a) 9.7–52.3%^b^ 1b) <2–42.6%^b^ *S. aureus* 2a) 1–39%^a^ 2b) 0.1–6%^a^ *Streptococcus equi* 2a) 4.5% (polyacrylic)^b^ 2b) No data without friction	Yes	With friction: Cotton textile and polyamide textile as destination (compared to other textiles) With friction: Polyacrylic textile as origin (compared to other synthetic textiles)
Gibson et al. (2012)^9^ Score: 60%	Murine Norovirus Feline calicivirus Bacteriophages PRD1 Bacteriophage MS2	Phosphate-buffered solution	1) Laminate 1) Stainless steel 2) Cellulose/cotton mix textile (moist) 2) Microfiber textile (moist) 2) Viscose/polyester mix textile (moist) 2) Cotton textile (moist)	1) Cellulose/cotton mix textile (moist) 1) Microfiber textile (moist) 1) Viscose/polyester mix textile (moist) 1) Cotton textile (moist) 2) Laminate 2) Stainless steel	Wiping (contact time or pressure not specified)	1) >99%^f^ 2) <1%^f^	Yes	For feline calicivirus: origin surface stainless steel (compared to laminate) Viscose/polyester textile and cotton textile as origin (compared to microfiber and cellulose/cotton textile)
Knobben et al. (2006)^11^ Score: 81%	*Staphylococcus aureus* *Staphylococcus epidermidis* *Propionibacterium acnes*	Saline 0.9%	1) Polyester textile (moist or dry) 2) Latex glove (moist or dry) 3) Stainless Steel (Broach moist or dry)	1a) Latex glove 1b) Stainless Steel 2, 3) Polyester textile	Pressure of 98kPa for 10s with and without friction	1a) 10–71%^a^ 1b) 11–32%^a^ 2) 14–80%^a^ 3) 20–67%^a^	Yes	Moisture Friction Stainless steel (low roughness and hydrophilic) as origin (compared to latex glove and polyester textile) Polyester textile (high roughness and hydrophobic) as destination (compared to latex glove and stainless steel)
Lopez et al. (2013)^26^ Score: 92%	*Escherichia coli* *Staphylococcus aureus* *Bacillus thuringiensis* MS2 coliphage	*E. coli and S. aureus*: Tryptic soy broth *B. thuringiensis*: Difco sporulation medium with supplements MS2 Coliphage: Tryptic soy broth and 3% beef extract	Cotton textile Polyester textile	Finger pad	1) Pressure of 98kPa for 10s (high environmental humidity) 2) Pressure of 98kPa for 10s (low environmental humidity)	1) Cotton textile: < 13.4% 1) Polyester textile: <0.7–5% 2) Cotton textile: <6.8% 2) Polyester textile: 0.3–<0.6%	Yes	Humidity
Mackintosh and Hoffman(1984)^17^ Score: 70%	*Staphylococcus saprophyticus* *Pseudomonas aeruginosa* *Escherichia coli* *Klebisella aerogenes* *Serratia marcescens* *Streptococcus pyogenes*	Nutrient broth	1) J-Cloth textile (exact material not specified) 2) Hand	1) Hand 2) J-Cloth textile	Pressure (grasp) for 10s (pressure not specified)	1) *S. saprophyticus*: 1.67% 1) All other strains: 0.01–0.47% 2) 17–88%	No	–
Mallick et al. (2021)^19^ Score: 67%	*Escherichia coli* *Staphylococcus aureus*	Glycerol stock in Luria Broth	1) Cotton textile 1) Polyester textile 1) Cotton/polyester mix textile 2) Surrogate skin	1) Surrogate skin 2) Cotton textile 2) Polyester textile 2) Cotton/polyester mix textile	Pressure of 16kPa for 10s with friction	1) 2–4% 2) 32–52%	Not described	Skin (low roughness) as origin (compared to all textiles) Polyester (low roughness) as origin (compared to other textiles) Friction
Marples and Towers (1979)^18^ Score: 67%	*Staphylococcus saprophyticus*	Nutrient broth or broth from overnight plate culture (not further specified)	1a) Viscose textile wet 1b) Viscose textile dry 2) Hand	1) Hand 2) Viscose textile	Pressure (grasp) (contact time or pressure not specified)	1a) 10% 1b) 0.05% 2) 85%	Yes	–
Moore and Griffith (2006)^12^ Score: 52%	*Staphylococcus aureus*	Tryptic soy broth and 5% horse serum	1) Stainless steel 2a) Microfiber textile wet 2b) Synthetic textile wet	1a) Synthetic textile wet 1b) Microfiber wet 2) Stainless steel	Wiping (contact time or pressure not specified)	1a) 96%^f^ 1b) 97.5%^f^ 2a) 0.1%^f^ 2b) 1.26%^f^	Not described	Moisture
Rusin et al. (2002)^25^ Score: 70%	*Serratia rubidaea* *Micrococcus luteus* PDR1-Phage	Tryptic soy broth	Cotton textile Cotton/polyester mix textile	Hand	1) Transferring a load laundry to dryer 2) Wringing out (contact time or pressure not specified)	1) <0.01–0.13 2) <0.01–0.04	Not described	–
Sattar et al. (2001)^5^ Score: 90%	*Staphylococcus aureus*	Tryptic soy broth and 5% fetal bovine serum	Cotton textile Cotton/polyester mix textile (dry or moist)	Finger pad Cotton textile Cotton/polyester mix textile (dry or moist)	Pressure of 20 kPa for 10s with and without friction	Dry origin to dry destination: 0.01–0.02%^a^ Dry origin to moist destination: 0.1–0.15%^a^ Moist origin to dry destination: 0.1–1% Moist origin to moist destination: 0.15–2.5%	Yes	Moisture Cotton/polyester mix textile as origin and destination (compared to cotton textile) Friction
Scott and Bloomfield (1990)^23^ Score: 65%	*Escherichia coli* *Klebisella aerogenes* *Staphylococcus aureus*	Tryptic soy broth diluted with distilled water	J-Cloth textile (exact material not specified)	1a) Finger 1b) Laminate	Finger pressed on textiles with 10 kPa for 30s Wiping with 20 kPa for 30s	1a) 0.5–6.7%^b^ 1b) 1.2–5.7%^b^	Yes	–
Sidwell et al. (1970)^10^ Score: 72%	1) Polio virus 2) Vaccinia virus	Cell culture suspension Stock	Cotton textile Wool textile Nylon textile Dacron/cotton mix textile	Destination was same as origin	Tumbling together up to 30min	1) Cotton textile: 2.5–63%^b^ 1) Wool textile: 6–32%^b^ 1) Nylon textile: 0.4–8%^b^ 1)Dacron/cotton mix textile: 25–32%^b^ 2) For all textiles: 0.2–8%^b^	Not relevant^c^	–
Smith et al. (2011)^13^ Score: 73%	*Clostridium difficile* *Escherichia coli* MRSA	Browne’s soil^e^	Stainless steel Ceramic tile Laminate	Microfiber textile	Wiping for 3.5s (pressure not specified)	95–99.9%^a^	Not described	*C. difficile* (compared to MRSA) *Escherichia coli (compared to* MRSA)
Trajtman et al. (2015)^14^ Score: 69%	*Clostridium difficile*	Artificial test soil	1) Ceramic tile 2a) Microfiber textile 2b) Cotton textile	1) Microfiber textile 1) Cotton textile 2) Ceramic tile	Wiping with 4 kPa (contact time not specified)	1) 98–99.4%^f^ 2) 0.05–0.17%^f^	Yes	Cotton textile as origin (vs. microfiber textile) For ceramic tiles: Destination surface microfiber textile (vs. cotton textile)
Varshney et al. (2020)^27^ Score: 77%	*Escherichia Coli* *Acinetobacter calcoaceticus*	Luria Bertani (LB) broth	Cotton textile Silk textile Viscose textile Wool textile Polyester textile Polypropylene textile Polyester-cotton (70:30%) textile (dry or moist)	Destination was same as origin (dry or moist)	Pressure of 196 kPa for 1 minute (static friction) Pressure of 20kPa by moving a pencil uniformly for 20 cycles (dynamic friction)	0.07–0.2%	Yes	Friction For *E. Coli and with friction*: Polypropylene textile (vs. other textiles) For *A. calcoaceticus and with friction*: Polyester-cotton textile (compared to other textiles)
Williams et al. (2007)^15^ Score: 69%	*Staphylococcus aureus* MSSA MRSA	Tryptone sodium chloride	Stainless steel	Polypropylen/viscose textile mix	Wiping with 1kPa for 10s	95–100%^f^	Not described	–

Note. MSSA, methicillin-susceptible Staphylococcus aureus; MRSA, methicillin-resistant Staphylococcus aureus; kPa, kilopascal.

a Results were extrapolated from Figures.

b Transfer percentage obtained via own calculations.

c Same origin and destination, same sampling technique on both origin and destination surface.

d Exposed to hexafluoroethane (C2F6) gas plasma.

e Defined artificial test soil used by National Health Service UK to validate equipment cleaning.

f Numerical values were extrapolated from Figures and transfer percentage was obtained via own calculations.

Twelve (57%) studies reported transfer proportions in the result section, with 5 (24%) studies only displaying transfer proportions in graphs and figures without reporting exact numbers. Calculations of transfer proportions from the authors of the present review were necessary in 9 (43%) studies. The most frequently investigated textile was cotton (13 studies and 103 experiments). *Staphylococcus aureus* was the most commonly investigated microorganism (13 studies and 109 experiments), followed by *Escherichia coli* (9 studies and 124 experiments). Two studies conducted transfer experiments with viruses.^9,10^ No experiments were published testing parasites or fungi. A wide variety of carrier materials for the microorganisms were used, with tryptone soy broth being the most frequent (6 studies and 103 experiments). Sampling methods were heterogeneous, with swabbing being the most frequently used (6 studies and 59 experiments). Only 10 studies reported on the recovery rate of the sampling method.

### Transfer proportions stratified by origin and destination materials

Origin materials were textiles, solid surfaces, and skin or “skin surrogates” such as artificial or pigskin, in sixteen, seven, and five studies, respectively. Destination materials were textiles, solid surfaces, and skin or skin surrogates in sixteen, five, and nine studies, respectively. One study used latex gloves as origin and destination material in textile transfer experiments.^11^

**Figure 2.**
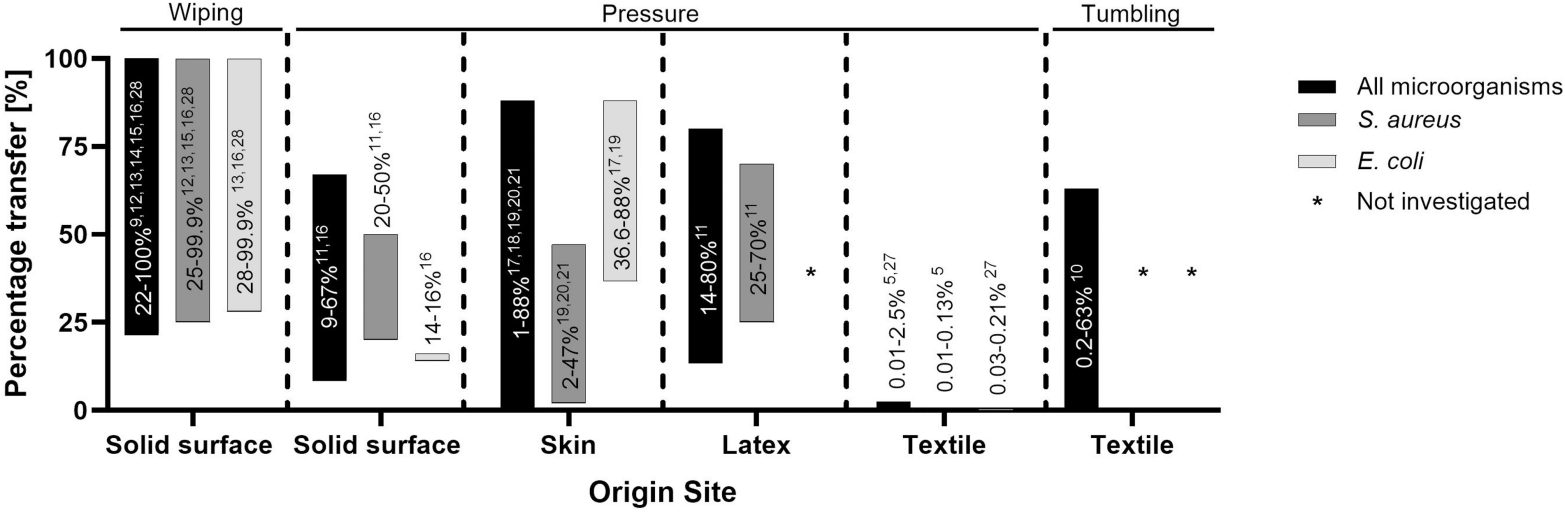
Transfer proportions from other materials to textiles. This figure shows the proportion of transfer from various tested materials to textiles. The y-axis represents the percentage of transfer, while the x-axis lists the material-to-textile combinations. The three bars indicate the transfer proportion for all microorganism, *S. aureus* and *E. coli*, with references to the studies that investigated each case. On top of each bar section the transfer mechanism is specified.

**Figure 3.**
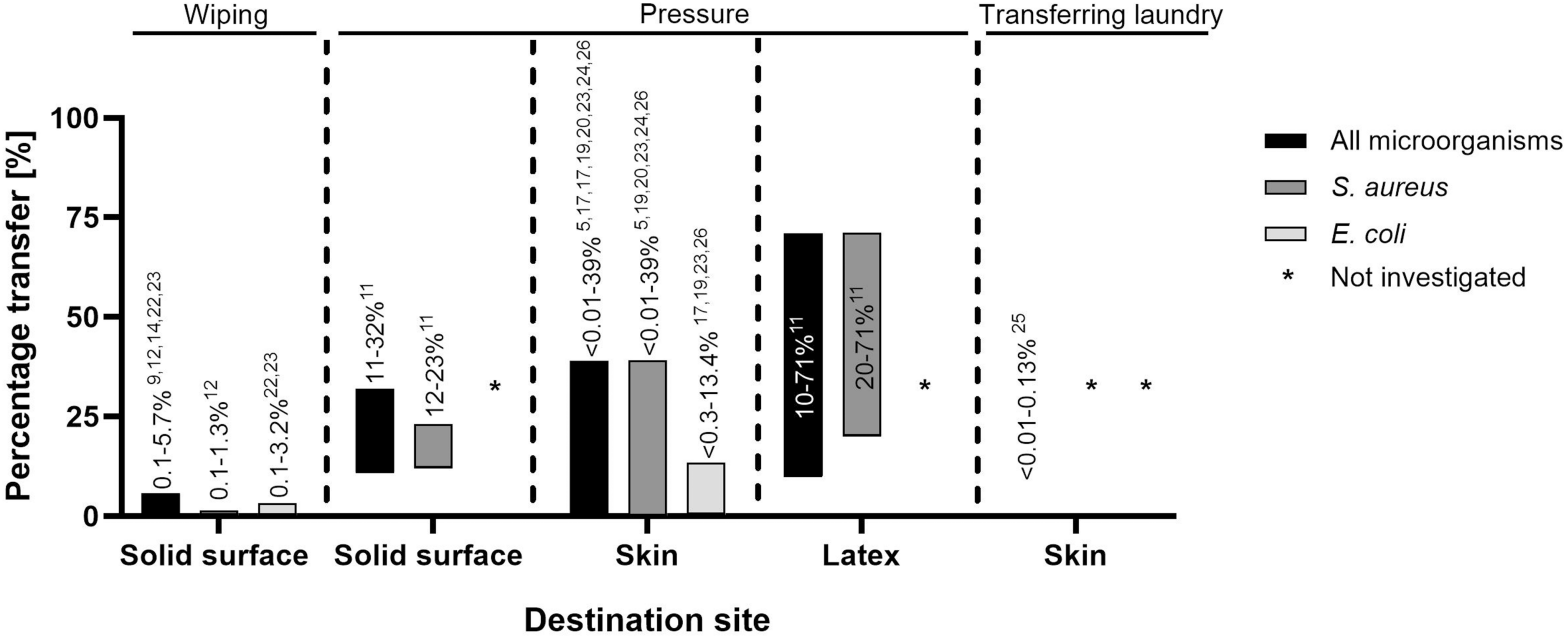
Transfer proportions from textiles to other materials. This figure shows the proportion of transfer from various tested textiles to other materials and textiles. The y-axis represents the percentage of transfer, while the x-axis lists the textile-to-material combinations. The three bars indicate the transfer proportion for all microorganism, *S. aureus* and *E. coli*, with references to the studies that investigated each case. On top of each bar section the transfer mechanism is specified.

Figure 2 illustrates experiments from other materials to textiles; Figure 3 illustrates experiments from textiles to other materials. Both figures include information on all microorganisms investigated, and for *S. aureus* and *E. coli* specifically.

#### Transfers from solid materials to textiles

Eight studies reported transfer from solid materials to textiles with transfer proportions from 9% to 100%. Transfer proportions of 85% to 100% were reported in experiments with transfer from an inoculated smooth solid material (ceramic tile, stainless steel, and laminate) to a textile cleaning cloth by wiping.^9,12–28^ Acrylic glass was the origin in one study, with lower transfer proportions of 22–53% by wiping.^16^ The transfer from solid materials to textiles by simple contact (pressure) was investigated by two studies, with transfer proportions between 9% and 16% for *E. coli, S. aureus*, and *E. faecalis* after pressure for 10 seconds,^16^ and between 20 and 67% for *S. epidermidis, S. aureus*, and *P. acnes* after pressure for 10 seconds with and without friction.^11^

#### Transfers from skin to textiles

Five studies investigated the transfer from skin or skin surrogates to textiles and reported highly variable transfer proportions from 1% to 88%. Proportions of 17–88% were reported in studies applying a grasping (pressure) action.^17,18^ In experiments applying pressure with friction, the transfer proportions ranged from 1% to 52%.^19,20^ In experiments with pressure only, proportions were mostly below 10%.^20,21^

#### Transfer from textiles to solid materials

Five of six studies that investigated the transfer from textiles to solid materials applied wiping. All five of these reported transfer proportions below 6%.^9,12,14,22,23^ The remaining study, which investigated transfer via pressure between polyester textile and stainless steel for 10 seconds with and without friction, reported a transfer proportion of 11–32% for gram-positive organisms.^11^

#### Transfer from textiles to skin

In the nine studies that investigated the transfer from textiles to skin or skin surrogates, transfer proportions ranged from <1% to 39%, with seven studies reporting transfer proportions of less than 10%.^5,17–19,23–25^ However, one study reported a transfer proportion of up to 39% for *S. aureus* and *S. equi* to artificial skin by pressure, and one study reported a transfer proportion of <13.4% when a finger pad contacted textile in high environmental humidity.^20,26^

#### Transfer between textiles

Three studies examined textile-to-textile transfers for *S. aureus*, Polio and Vaccinia viruses, and *Acinetobacter calcoaceticus* and *E. coli*, respectively.^5,10,27^ Pressure transfer was up to 2.5% for *S. aureus*, and up to 0.2% for *E. coli* and *A. calcoaceticus*. Polio and vaccinia virus transfer varied from <1% to 63% when textiles were tumbled together.

### Factors influencing transfer proportion

Thirteen studies examined how factors such as material, moisture, transfer mechanism, and type of microorganism influence the transfer of microorganisms.

#### Origin and destination material

Several studies reported variations in microorganism transfer depending on the textile. Three studies reported that synthetic cloths such as polyester, polyacrylic, and polyamide cloths had higher transfer proportions than pure cotton cloth.^5,19,27^ Microfiber cloths undergoing several washing cycles were reported to remove microorganisms better than new microfiber cloths.^13^ One study reported conflicting results with better removal of microorganisms from reprocessed cotton cloths compared to reprocessed microfiber cloths.^28^

The transfer from surrogate skin to textile was reported to be almost 10 times higher than reciprocally.^19^ Cotton/polyester blend textile were described to be both better donor and recipient material than pure cotton textile.^5^ Contrary to these findings, another study observed a greater transfer proportion to cotton textiles than to polyester textiles.^20^ In a separate study, which investigated a different microorganism and compared polypropylene and lyocell textile, higher transfer proportions were reported to polypropylene and hexafluoroethane-treated lyocell textile than to untreated lyocell textile.^16^

#### Moisture and humidity

All four studies investigating the effect of moisture reported increased transfer proportions with increasing moisture.^5,11,12,28^ This was true for both textiles and other materials and whether materials were origin^5,11^ or destination.^12,28^ One study reported two to threefold higher transfer proportions when the original material was moistened, compared to experiments in dry conditions.^11^ Moistening both origin and destination materials resulted in the highest transfer proportions compared to experiments where materials were dry.^5^

Similarly, transfer proportions increased when transfer was performed in high relative environmental humidity, without moistening.^21,26^

#### Action leading to transfer

Six studies reporting differences of transfer mechanisms found that the application of friction, or dynamic wiping, in comparison to static wiping, resulted in higher transfer proportions,^5,11,16,19,20,27^ quantified as increase of 5–61%^27^ or by a factor of five.^5^

#### Microorganism

One study described that *E. coli* was transferred more easily compared to Gram-positive bacteria such as *S. aureus* and *E. faecalis* from skin to synthetic fibers.^16^ Similar results were reported by others, where *E. coli* was more easily transferred compared to methicillin-resistant Staphylococcus aureus (MRSA).^13^ Others found no statistically significant difference between *E. coli* and *S. aureus*, whether transferred from skin to textile or reciprocally.^19^ Appendix Tables 3 and 4 provide detailed data on specific textiles and the transfer proportions of *S. aureus and E. coli*.

Wiping of solid material with textiles, independently from the textile, resulted in lower removal of *Murine norovirus* compared to other viruses (*Feline calicivirus* and *Bacteriophages*).^9^

### Quality of the included studies

The mean study quality score of the modified Downs and Black Checklist was 73% and ranged from 52 to 92% (Appendix Table 5). The most common reasons for lower scores included failing to report estimates of random variability in transfer proportions (*n* = 18, 86%), environmental conditions (*n* = 16, 76%), and results of transfer proportions as percentages (*n* = 9, 43%).

## Discussion

This systematic review summarizes the existing literature on the transfer of microorganisms from and to textiles. We found considerable heterogeneity for all relevant study variables such as the origin and destination material, investigated pathogens, carrier materials, the origin and destination surface, and transfer mechanisms. This heterogeneity resulted in a broad range of reported transfer proportions from less than 1% to up to 100%. A few key factors associated with transfer of microorganisms were identified such as moisture, application of friction, and specific types of textiles.

In the hospital context, two types of textiles can be distinguished: materials to absorb microorganisms such as cleaning cloths, and materials to resist contamination with microorganisms such as bedding or clothing. Weaving patterns, density as well as materials roughness affect bacterial binding and disposal.^27,29^ Our review found that synthetic textiles, particularly polyester and similar compounds, transfer bacteria more easily than cotton.^5,19,27^ Roughness plays a key role here, with smooth materials facilitating transfer. Polyester is a particularly smooth material, followed by polyester compounds, while cotton, or polypropylene (commonly used in isolation gowns) is rougher.^19,27^ Synthetic textiles, such as polyester, may have enhanced transfer proportions owing to their elevated coefficient of friction (i.e. representing the force needed to move one surface over another) and hydrophobic characteristics, both of which are associated with improved transfer efficiency to and from materials.^19,30^ This aligns with research indicating that bacteria preferentially attach to surfaces that resemble their own surface energy, structure, and hydrophobic characteristics.^16^ Type and physical properties of the textile material play an important role and can either prevent or facilitate the transfer of microorganisms. When comparing the results on the proportion of microorganisms transferred to textiles, our literature review found contradicting results. While one study reported higher transfer to a cotton/polyester blend than to cotton, another found greater transfer to cotton than to polyester.^5,20^

Humidity and moistening of surfaces are important determinants. Transfer of microorganisms from and to textiles increases with the presence of moisture on either the origin or destination material. Low humidity affects microbial growth, metabolism, and survival, causing shrinkage and suppressing replication, which all may reduce microbial transfer.^31^ In daily practice, transfer is thus more likely to occur from or to moist textiles such as towels or shower curtains. Also, material contaminated with body fluids are more likely to facilitate the transmission of microorganisms. While handling of body fluids is perceived a risk by most healthcare workers and appropriate hand hygiene measures are recommended,^32^ the risk from wet towels, cloths, or shower drains likely often is underestimated.

The application of friction was another factor that increased the transfer of microorganisms. Friction promotes transfer by mechanically breaking hydrophobic bonds, van der Waals forces, and hydrogen bonds.^19^ Friction is used intentionally in cleaning or hand drying with towels but also unintentionally in touching patients for mobilization or repositioning or firmly grasping textiles, e.g. privacy curtains. Based on our findings, all these actions are associated with increased transfer risk. Brief, dry, non-frictional contacts such as lightly touching a patient’s bedding, may have a lower transfer potential. Awareness of such specific risks can help guide infection control practices.

Our systematic literature review had several limitations. First, we could not conduct a meta-analysis due to heterogeneity of the included studies. Differences concerned experimental setups, sampling methods, and the reporting of outcomes. We still summarized the existing evidence by grouping studies with similar features to allow data synthesis to be structured, transparent, and focused. Second, the fact that only about half of the included studies investigated the recovery rate of their sampling methods introduces potential bias to the reported transfer proportions. This highlights the need for standardized protocols in future research for reliable and comparable data. Finally, several studies only displayed transfer proportions in figures without reporting numerical data or did not report transfer proportions at all. Transfer proportions were then calculated by the systematic review team based on the reported raw data. However, this introduced a potential source of error. We therefore clearly indicated studies in which results were extrapolated or self-calculated.

In conclusion, this review highlights the complexity of microbial transfer between textiles and other materials, influenced by a multitude of factors including textile material, transfer mechanism, and environmental conditions. While the risk of microbial transfer by a short non-frictional contact of textile with other material is low, the presence of humidity and friction increases the likelihood of transmission considerably. The result of this review informs future guidelines on the use of personal protective equipment such as gowns or aprons, on hospital laundry policies, and on the selection of textiles in healthcare.

## Supporting information

Gassmann et al. supplementary material 1 Gassmann et al. supplementary material

Gassmann et al. supplementary material 2 Gassmann et al. supplementary material

Gassmann et al. supplementary material 3 Gassmann et al. supplementary material

Gassmann et al. supplementary material 4 Gassmann et al. supplementary material

Gassmann et al. supplementary material 5 Gassmann et al. supplementary material

## Supplementary material

To view supplementary material for this article, please visit https://doi.org/10.1017/ice.2025.10299.

## References

[1] Otter JA , Yezli S , French GL. The role played by contaminated surfaces in the transmission of nosocomial pathogens. Infect Control Hosp Epidemiol 2011;32:687–99. 21666400 10.1086/660363

[2] Mitchell A , Spencer M , Edmiston C, Jr . Role of healthcare apparel and other healthcare textiles in the transmission of pathogens: a review of the literature. J Hosp Infection 2015;90:285–92. 10.1016/j.jhin.2015.02.017 PMC7132459 25935701

[3] Ohl M , Schweizer M , Graham M , Heilmann K , Boyken L , Diekema D. Hospital privacy curtains are frequently and rapidly contaminated with potentially pathogenic bacteria. Am J Infect Control 2012;40:904–6. 22464039 10.1016/j.ajic.2011.12.017

[4] Neely AN. A survey of gram-negative bacteria survival on hospital fabrics and plastics. J Burn Care Rehabilitation 2000;21:523–7. 10.1097/00004630-200021060-00009 11194806

[5] Sattar SA , Springthorpe S , Mani S , Gallant M , Nair RC , Scott E , et al. Transfer of bacteria from fabrics to hands and other fabrics: development and application of a quantitative method using Staphylococcus aureus as a model. J Appl Microbiol 2001;90:962–70. 11412326 10.1046/j.1365-2672.2001.01347.x

[6] Kampf G. How long can nosocomial pathogens survive on textiles? A systematic review. GMS Hygiene and Infect Control 2020;15:Doc10. 10.3205/dgkh000345 PMC7273332 32547910

[7] Owen L , Laird K. The role of textiles as fomites in the healthcare environment: a review of the infection control risk. PeerJ 2020;8:e9790. 32904371 10.7717/peerj.9790 PMC7453921

[8] Downs SH , Black N. The feasibility of creating a checklist for the assessment of the methodological quality both of randomised and non-randomised studies of health care interventions. J Epidemiol Community Health 1998;52:377–84. 9764259 10.1136/jech.52.6.377 PMC1756728

[9] Gibson KE , Crandall PG , Ricke SC. Removal and transfer of viruses on food contact surfaces by cleaning cloths. Appl Environ Microbiol 2012;78:3037–44. 22327573 10.1128/AEM.00027-12 PMC3346440

[10] Sidwell RW , Dixon GJ , Westbrook L , Forziati FH. Quantitative studies on fabrics as disseminators of viruses. IV. Virus transmission by dry contact of fabrics. Appl Microbiol 1970;19:950–4. 4318451 10.1128/am.19.6.950-954.1970 PMC376831

[11] Knobben BA, van der Mei Hc Fau - van Horn JR, van Horn Jr Fau - Busscher HJ, Busscher HJ. Transfer of bacteria between biomaterials surfaces in the operating room-an experimental study. 2007(1549-3296 (Print)). 10.1002/jbm.a.30978 17058211

[12] Moore G , Griffith C. A laboratory evaluation of the decontamination properties of microfibre cloths. J Hosp Infection 2006;64:379–85. 10.1016/j.jhin.2006.08.006 17055112

[13] Smith DL , Gillanders S , Holah JT , Gush C. Assessing the efficacy of different microfibre cloths at removing surface micro-organisms associated with healthcare-associated infections. J Hosp Infection 2011;78:182–6. 10.1016/j.jhin.2011.02.015 21501897

[14] Trajtman AN , Manickam K , Alfa MJ. Microfiber cloths reduce the transfer of Clostridium difficile spores to environmental surfaces compared with cotton cloths. Am J Infect Control 2015;43:686–9. 25907782 10.1016/j.ajic.2015.03.002

[15] Williams GJ , Denyer SP , Hosein IK , Hill DW , Maillard JY. The development of a new three-step protocol to determine the efficacy of disinfectant wipes on surfaces contaminated with Staphylococcus aureus. J Hosp Infection 2007;67:329–35. 10.1016/j.jhin.2007.08.012 17945392

[16] Edwards NWM , Best EL , Connell SD , Goswami P , Carr CM , Wilcox MH , et al. Role of surface energy and nano-roughness in the removal efficiency of bacterial contamination by nonwoven wipes from frequently touched surfaces. Sci Technol Adv Mater 2017;18:197–209. 28469734 10.1080/14686996.2017.1288543 PMC5404180

[17] Mackintosh CA , Hoffman PN. An extended model for transfer of micro-organisms via the hands: differences between organisms and the effect of alcohol disinfection. J Hyg (Lond) 1984;92:345–55. 6429238 10.1017/s0022172400064561 PMC2129313

[18] Marples RR , Towers AG. A laboratory model for the investigation of contact transfer of micro-organisms. J Hyg (Lond) 1979;82:237–48. 429788 10.1017/s0022172400025651 PMC2130140

[19] Mallick D , Gupta D , Sharma S. Transfer of bacteria between fabric and surrogate skin. Am J Infect Control 2022;50:758–763. 34774893 10.1016/j.ajic.2021.10.040

[20] Gerhardts A , Henze SV , Bockmuhl D , Hofer D. Fabric-skin models to assess infection transfer for impetigo contagiosa in a kindergarten scenario. Eur J Clin Microbiol Infect Dis 2015;34:1153–60. 25666081 10.1007/s10096-015-2336-7

[21] Arinder P , Johannesson P , Karlsson I , Borch E. Transfer and decontamination of s. aureus in transmission routes regarding hands and contact surfaces. PLoS ONE [Electronic Resource]. 2016;11:e0156390. 27280772 10.1371/journal.pone.0156390 PMC4900614

[22] Bartz S , Ritter AC , Tondo EC. Evaluation of bacterial multiplication in cleaning cloths containing different quantities of organic matter. J Infect Dev Countries 2010;4:566–71. 10.3855/jidc.689 21045369

[23] Scott E , Bloomfield SF. The survival and transfer of microbial contamination via cloths, hands and utensils. J Appl Bacteriol 1990;68:271–8. 2111304 10.1111/j.1365-2672.1990.tb02574.x

[24] Desai R , Pannaraj PS , Agopian J , Sugar CA , Liu GY , Miller LG. Survival and transmission of community-associated methicillin-resistant Staphylococcus aureus from fomites. Am J Infect Control 2011;39:219–25. 21458684 10.1016/j.ajic.2010.07.005

[25] Rusin P , Maxwell S , Gerba C. Comparative surface-to-hand and fingertip-to-mouth transfer efficiency of gram-positive bacteria, gram-negative bacteria, and phage. J Appl Microbiol 2002;93:585–92. 12234341 10.1046/j.1365-2672.2002.01734.x

[26] Lopez GU , Gerba CP , Tamimi AH , Kitajima M , Maxwell SL , Rose JB. Transfer efficiency of bacteria and viruses from porous and nonporous fomites to fingers under different relative humidity conditions. Appl Environ Microbiol 2013;79:5728–34. 23851098 10.1128/AEM.01030-13 PMC3754157

[27] Varshney S , Pandey P , Gupta D , Sharma S. Role of fabric properties, moisture and friction in transfer of bacteria from fabric to fabric. Text Res J 2020;90:478–85.

[28] Diab-Elschahawi M , Assadian O , Blacky A , Stadler M , Pernicka E , Berger J , et al. Evaluation of the decontamination efficacy of new and reprocessed microfiber cleaning cloth compared with other commonly used cleaning cloths in the hospital. Am J Infect Control 2010;38:289–92. 20123151 10.1016/j.ajic.2009.09.006

[29] Bakterij A. An overview of the influence of stainless-steel surface properties on bacterial adhesion. Mater Tehnol 2014;48:609–17.

[30] Møllebjerg A , Palmén LG , Gori K , Meyer RA-O. The Bacterial Life Cycle in Textiles is Governed by Fiber Hydrophobicity. 2021(2165-0497 (Electronic)). 10.1128/Spectrum.01185-21 PMC8515937 34643452

[31] Qiu Y , Zhou Y , Chang Y , Liang X , Zhang H , Lin X , et al. The effects of ventilation, humidity, and temperature on bacterial growth and bacterial genera distribution. Int J Environ Res Public Health 2022;19:15345. 36430064 10.3390/ijerph192215345 PMC9691097

[32] No authors listed. World Health Organization. Five moments for hand hygiene. 2021. Available from: https://www.who.int/publications/m/item/five-moments-for-hand-hygiene (last accessed 7 April 2025).

