# Supplementary material for: Transfer of microorganisms to and from textiles in healthcare settings: a systematic review
Source: Infect Control Hosp Epidemiol. 2025 Oct 16;46(12):1243–52. doi: 10.1017/ice.2025.10299 (PMC12779459; doi:10.1017/ice.2025.10299)
Supplement: Gassmann et al. supplementary material 2 — Gassmann et al. supplementary material [file S0899823X25102997sup002.docx]

# Appendix - Supplementary material

**Appendix Table 2.** Assessment of study quality

The quality assessment includes 12 items, with 1 point assigned to a study that meets the quality criterium, and 0 points assigned to a study that does not meet the quality criterium. Exception is quality item number 7 (in modified checklist) that assigns 1 point for meeting item 7a, 0.75 points for meeting item 7b, 0.5 points for meeting item 7c, and 0.25 points for meeting item 7d.

The maximum quality score is 12 points, equivalent to 100%. However, for studies where some quality items were marked as not applicable (n.a.), the maximum possible score was adjusted accordingly. The reason for non-applicability is provided in the footnotes of appendix table 2.

**Bold text** highlights the necessary modifications to adapt the quality assessment for the non-interventional studies included in our review. Items deemed not applicable (n.a.) to these studies were excluded from the quality assessment but are listed in the table.

| **Item in B&B Checklist** | **Original quality items by Downs and Black** | **Item in modified checklist** | **Adapted quality items for this study** | **Studies meeting quality item (%)** |
| --- | --- | --- | --- | --- |
|  | **Reporting** | | | |
| 1 | Is the hypothesis/aim/objective of the study clearly described? | 1 | Is the hypothesis/aim/objective of the study clearly described? | 21/21 (100) |
| 2 | Are the main outcomes to be measured clearly described in the Introduction or Methods section? | 2 | Was the transfer percentage a defined study outcome? | 16/21 (76) |
| 3 | Are the characteristics of the patients included in the study clearly described? | **3** | **Are the environmental conditions in which the transfer experiment took place** clearly described? | 4/22 (19) |
| 4 | Are the interventions of interest clearly described? | **4** | **Are the actions leading to transfer clearly described**? | 21/21 (100) |
| 4 |  | **5** | **Are the sampling and microbiological culturing methods** clearly described? | 20/21 (95) |
| 4 |  | 6 | Was the sampling method **validated concerning recovery**? | 12/21 (57)^a b^ |
| 5 | Are the distributions of principal confounders in each group of subjects to be compared clearly described? |  | n.a. |  |
| 6 | Are the main findings of the study clearly described? | 7a | **Does the study provide numerical results about transfer percentage**? | 8/21 (38)* |
| 6 |  | **7b** | **If no, does the study provide graphical results about transfer percentage?** | 3/21 (14) |
| 6 |  | **7c** | **If no, does the study provide numerical results that allow own calculation of transfer percentage?** | 10/21 (48)* |
| 6 |  | **7d** | **If no, does the study provide graphical results that allow own calculation of transfer percentage?** | 1/21 (5) |
| 7 | Does the study provide estimates of the random variability in the data for the main outcomes? | 8 | Does the study provide estimates of the random variability in the data for transfer percentage? | 2/21 (10) |
| 8 | Have all important adverse events that may be a consequence of the intervention been reported? |  | n.a. |  |
| 9 | Have the characteristics of patients lost to follow-up been described? |  | n.a. |  |
| 10 | Have actual probability values been reported (e.g. 0.035 rather than <0.05) for the main outcomes except where the probability value is less than 0.001? | **9** | **In studies assessing the impact of independent factors on the transfer percentage,** have actual probability values been reported (e.g. 0.035 rather than <0.05) for the analysis except where the probability value is less than 0.001? | 9/21 (43) |
|  | **External validity** | | | |
| 11 | Were the subjects asked to participate in the study representative of the entire population from which they were recruited? |  | n.a. |  |
| 12 | Were those subjects who were prepared to participate representative of the entire population from which they were recruited? |  | n.a. |  |
| 13 | Were the staff, places, and facilities where the patients were treated, representative of the treatment the majority of patients receive? | **10** | **Are the textiles used in the healthcare setting and are transfer actions happening in the healthcare setting?** | 21/21 (100) |
|  | **Internal validity - bias** | | | |
| 14 | Was an attempt made to blind study subjects to the intervention they have received? |  | n.a. |  |
| 15 | Was an attempt made to blind those measuring the main outcomes of the intervention? |  | n.a. |  |
| 16 | If any of the results of the study were based on “data dredging”, was this made clear? |  | n.a. |  |
| 17 | In trials and cohort studies, do the analyses adjust for different lengths of follow-up of patients, or in case-control studies, is the time period between the intervention and outcome the same for cases and controls? |  | n.a. |  |
| 18 | Were the statistical tests used to assess the main outcomes appropriate? | 11 | Were the statistical tests used to assess **the impact of independent factors on transfer percentages** appropriate? | 16/21 (76) |
| 19 | Was compliance with the intervention/s reliable? | 12 | Was compliance with the **action to transfer the microorganisms reliable**? | 21/21 (100) |
| 20 | Were the main outcome measures used accurate (valid and reliable)? |  | n.a. |  |
|  | **Internal validity - confounding** | | | |
| 21 | Were the patients in different intervention groups (trials and cohort studies) or were the cases and controls (case-control studies) recruited from the same population? |  | n.a. |  |
| 22 | Were study subjects in different intervention groups (trials and cohort studies) or were the cases and controls (case-control studies) recruited over the same period of time? |  | n.a. |  |
| 23 | Were study subjects randomised to intervention groups? |  | n.a. |  |
| 24 | Was the randomised intervention assignment concealed from both patients and health care staff until recruitment was complete and irrevocable? |  | n.a. |  |
| 25 | Was there adequate adjustment for confounding in the analyses from which the main findings were drawn? |  | n.a. |  |
| 26 | Were losses of patients to follow-up taken into account? |  | n.a. |  |
|  | **Power** | | | |
| 27 | Did the study have sufficient power to detect a clinically important effect where the probability value for a difference being due to chance is less than 5%? |  | n.a. |  |

*One study (Gerhards et al.) provided the transfer percentage for one strain, for the other strain own calculations were necessary.

^a^ In one study (Sidwell et al.) recovery was unnecessary because origin and destination were the same, this was interpreted as “fulfilled criterium”.

^b^ One study (Scott and Bloomfield) did not report recovery for every strain tested, this was interpreted as “fulfilled criterium”
