# Supplementary material for: Transfer of microorganisms to and from textiles in healthcare settings: a systematic review
Source: Infect Control Hosp Epidemiol. 2025 Oct 16;46(12):1243–52. doi: 10.1017/ice.2025.10299 (PMC12779459; doi:10.1017/ice.2025.10299)
Supplement: Gassmann et al. supplementary material 3 — Gassmann et al. supplementary material [file S0899823X25102997sup003.docx]

**Appendix Table 3.** Transfer proportion overview by textile type for *S. aureus* (including MRSA).

| Origin | Destination | Action | Transfer proportion | Studied in author (year) | n= number of experiments (Studies) |
| --- | --- | --- | --- | --- | --- |
| Skin | Cotton textile | Pressure | 2-32% | Arinder (2016)  Gerhardts (2015)  Mallick (2021) | 6 (19, 20, 21) |
| Skin | Polyester-Cotton 50/50% textile | Pressure | 37% | Mallick (2021) | 1 (19) |
| Skin | Polyester textile | Pressure | 47.2 | Mallick (2021) | 1 (19) |
| Solid surface | Cotton textile | Wiping | 98-99.9% | Diab-Elschahawi (2010) | 2 (28) |
| Solid surface | Microfiber textile | Wiping | 96-99.9% | Diab-Elschahawi (2010) Moore and Griffith (2006) Smith (2011) | 3 (12, 13, 28) |
| Solid surface | Polyester textile | Pressure | 20-50% | Knobben (2006) | 3 (11) |
| Solid surface | Polyprpylene/viscose textile | Wiping | 96-98.4% | Williams (2007) | 3 (15) |
| Solid surface | Synthetic textile | Wiping | 96% | Moore and Griffith (2006) | 1 (12) |
| Acrylic glass | Lyocell textile | Pressure | 9-11% | Edwards (2017) | 3 (16) |
| Acrylic glass | Polypropylene | Pressure | 13% | Edwards (2017) | 1 (16) |
| Acrylic glass | Lyocell textile | Wiping | 25-33% | Edwards (2017) | 3 (16) |
| Acrylic glass | Polypropylene textile | Wiping | 35% | Edwards (2017) | 1 (16) |
| Cotton textile | Cotton textile | Pressure | 0.01-0.03% | Sattar (2001) | 3 (5) |
| Polyester-Cotton 50/50% textile | Polyester-Cotton 50/50% textile | Pressure | 0.02-0.13% | Sattar (2001) | 4 (5) |
| Latex | Polyester textile | Pressure | 25-70% | Knobben (2006) | 3 (11) |
| Cotton textile | Skin | Pressure | 0.01-9% | Desai (2011) Gerhardts (2015) Lopez (2013) Mallick (2021) Sattar (2001) | 15 (5, 19, 20, 24, 26) |
| Polyacrylic textile | Skin | Pressure | 3-39% | Gerhardts (2015) | 2 (20) |
| Polyester textile | Skin | Pressure | <0.3-12% | Gerhardts (2015) Lopez (2013) Mallick (2021) | 5 (19, 20, 26) |
| Polyester-Cotton 50/50% textile | Skin | Pressure | 0.01-2.5% | Mallick (2021) Sattar (2001) | 19 (5, 19) |
| Polyester textile | Latex | Pressure | 20-71% | Knobben (2006) | 3 (11) |
| Polyester textile | Solid surface | Pressure | 12-23% | Knobben (2006) | 2 (11) |
| Microfiber textile | Solid surface | Wiping | 0.1% | Moore and Griffith (2006) | 1 (12) |
| Synthetic textile | Solid surface | Wiping | 1.3% | Moore and Griffith (2006) | 1 (12) |
