# Supplementary material for: Transfer of microorganisms to and from textiles in healthcare settings: a systematic review
Source: Infect Control Hosp Epidemiol. 2025 Oct 16;46(12):1243–52. doi: 10.1017/ice.2025.10299 (PMC12779459; doi:10.1017/ice.2025.10299)
Supplement: Gassmann et al. supplementary material 4 — Gassmann et al. supplementary material [file S0899823X25102997sup004.docx]

**Appendix** **Table 4.** Transfer proportion overview by textile type for *E.coli.*

| Origin | Destination | Action | Transfer proportion | Studied in author (year) | n= number of experiments (Studies) |
| --- | --- | --- | --- | --- | --- |
| Cotton textile | Solid surface | Wiping | 2.1–2.6% | Bartz (2010) | 1 (22) |
| Solid surface | Cotton textile | Wiping | 97.4-99.9% | Diab-Elschahawi (2010) | 2 (28) |
| Solid surface | Microfiber textile | Wiping | 97.5-99.9% | Diab-Elschahawi (2010)  Smith (2011) | 12 (13, 28) |
| Acrylic glass | Lyocell textile | Wiping | 28-52% | Edwards (2017) | 6 (16) |
| Acrylic glass | Lyocell textile | Pressure | 14-15% | Edwards (2017) | 3 (16) |
| Acrylic glass | Polypropylene textile | Wiping | 53% | Edwards (2017) | 1 (16) |
| Acrylic glass | Polypropylene textile | Pressure | 16% | Edwards (2017) | 1 (16) |
| Cotton textile | Skin | Pressure | 2-13.4% | Lopez (2013)  Mallick (2021) | 3 (19, 26) |
| Polyester textile | Skin | Pressure | <0.3-4% | Lopez (2013) Mallick (2021) | 3 (19, 26) |
| Cellulose textile | Skin | Grasp (pressure) | 0.36% | Mackintosh and Hoffman (1984) | 1 (17) |
| Polyester-Cotton 50/50% textile | Skin | Pressure | 2.5% | Mallick (2021) | 1 (19) |
| J-textile | Skin | Pressure | 0.5% | Scott and Bloomfield (1990) | 1 (23) |
| J-textile | Solid surface | Wiping | 3.2% | Scott and Bloomfield (1990) | 1 (23) |
| Skin | Cellulose textile | Grasp (pressure) | 88% | Mackintosh and Hoffman (1984) | 1 (17) |
| Skin | Cotton | Pressure | 36.6% | Mallick (2021) | 1 (19) |
| Skin | Polyester-Cotton 50/50% textile | Pressure | 44.5% | Mallick (2021) | 1 (19) |
| Skin | Polyester textile | Pressure | 51.7% | Mallick (2021) | 1 (19) |
| Cotton textile | Cotton textile | Pressure | 0.07-0.11% | Varshney (2020) | 12 (27) |
| Polyester Cotton blend (70:30%) textile | Polyester Cotton blend (70:30%) textile | Pressure | 0.05-0.1% | Varshney (2020) | 12 (27) |
| Polyester textile | Polyester textile | Pressure | 0.07.0.21% | Varshney (2020) | 12 (27) |
| Polypropylene textile | Polypropylene textile | Pressure | 0.03-0.12% | Varshney (2020) | 12 (27) |
| Silk textile | Silk textile | Pressure | 0.03-0.08% | Varshney (2020) | 12 (27) |
| Viscose textile | Viscose textile | Pressure | 0.1-0.18% | Varshney (2020) | 12 (27) |
| Wool textile | Wool textile | Pressure | 0.08-0.12 | Varshney (2020) | 12 (27) |
